# Eosinophil-Derived Neurotoxin Is Elevated in Patients with Amyotrophic Lateral Sclerosis

**DOI:** 10.1155/2013/421389

**Published:** 2013-02-20

**Authors:** Guan-Ting Liu, Chi-Shin Hwang, Chia-Hung Hsieh, Chih-Hao Lu, Sunny Li-Yun Chang, Jin-Ching Lee, Chien-Fu Huang, Hao-Teng Chang

**Affiliations:** ^1^Graduate Institute of Molecular Systems Biomedicine, College of Medicine, China Medical University, Taichung 40402, Taiwan; ^2^Graduate Institute of Basic Medical Science and Ph.D. Program for Aging, China Medical University, No. 91 Hsueh-Shih Road, Taichung 40402, Taiwan; ^3^Department of Neurology, Taipei City Hospital, Zhongxiao Branch, Taipei 11556, Taiwan; ^4^Department of Medical Research, China Medical University Hospital, Taichung 40402, Taiwan; ^5^Department of Biotechnology, Kaohsiung Medical University, Kaohsiung 80708, Taiwan; ^6^Department of Biological Science and Technology, I-Shou University, Kaohsiung 82445, Taiwan

## Abstract

*Background and Objectives*. Amyotrophic lateral sclerosis (ALS) is a progressive neurodegenerative disease characterized by loss of motor neurons in the brainstem, motor cortex, and spinal cord. Oxidative stress and neuroinflammation have been implicated in the pathophysiology of ALS. Members of the family of damage-associated molecular patterns, including reactive oxygen species, high-mobility group box 1, and eosinophil-derived neurotoxin (EDN), may participate in pathological conditions. In this study, we aim to discover new biomarker for detecting ALS. *Materials and Methods*. We examined 44 patients with ALS, 41 patients with Alzheimer's disease, 41 patients with Parkinson's disease, and 44 healthy controls. The concentration of serum EDN was measured using an enzyme-linked immunosorbent assay. *Results*. EDN levels were significantly increased 2.17-fold in the serum of patients with ALS as compared with healthy controls (*P* < 0.05). No correlation between the levels of serum EDN and various clinical parameters of ALS was found. Moreover, the levels of serum EDN in patients with Parkinson's disease and Alzheimer's disease and healthy controls were similar. *Conclusion*. A higher level of serum EDN was found specifically in patients with ALS, indicating that EDN may participate in the pathophysiology of ALS.

## 1. Introduction

Amyotrophic lateral sclerosis (ALS) is the most common and devastating adult-onset neurodegenerative disease [1]. The underlying pathology involves the selective loss of motor neurons in the spinal cord, brainstem, and cerebral cortex [2]. Weakness and muscle atrophy are the typical earliest symptoms of ALS and are followed by rapid progression leading to total paralysis and respiratory failure within 2 to 5 years after diagnosis. Generally, ALS is considered to be a neuromuscular disorder, but more studies are beginning to recognize ALS as a multisystem neurological disease [3–5]. The annual incidence of ALS is 1-2 per 100,000 persons [6]. There are two types of ALS. One is familial ALS, which accounts for only 10% of all ALS cases [7]. A known hereditary factor, mutant Cu/Zn superoxide dismutase 1 (SOD1), is associated with ~20% of cases of familial ALS. The second type of ALS is sporadic ALS, the cause(s) of which is less well understood.

Currently, the etiology of ALS is unclear. However, several lines of evidence suggest that neuroinflammation [8], glutamate excitotoxicity [9], altered cytoskeletal proteins [10], impaired axoplasmic transport [11], and oxidative stress [12] are involved in disease development. Oxidative stress is believed to play an important role in ALS. Some SOD1 mutations such as SOD1-G93A, SOD1-G85R, and SOD1-G37R lead to a loss of dismutase activity in transgenic mice, resulting in the accumulation of high concentrations of reactive oxygen species (ROS) in motor neurons [13]. Subsequently, the free radicals generated by superoxide may damage neurons. Moreover, as motor neurons encounter excessive oxidative stress, high levels of cytoprotective heat shock proteins (HSPs) may be induced [14]. Generally, overexpression of HSPs is associated with cellular stress responses including heat shock [15], heavy metal stress [16], and disease [17]. A recent ALS study has indicated that the serum levels of HSPs are elevated in patients with ALS and in ALS mouse models [14]. Expression of protective proteins suggests that ALS may result from pathophysiological stress such as neuroinflammation. Neuroinflammation has been widely discussed as a mechanism of ALS [18]. During chronic neuroinflammation, activated microglia play direct and indirect destructive roles in inducing the expression and release of cytokines such as tumor necrosis factor-*α*, which stimulates local inflammatory responses and neuronal degeneration [19, 20]. Furthermore, microglia also secrete proinflammatory factors such as chemokines and ROS, which contribute to neuroinflammatory processes [21, 22]. Microglia may attack neurons, inducing progressive cell loss in specific neuronal populations in neurodegenerative disorders [23–25]. Therefore, factors that induce inflammatory responses could serve as potential biomarkers. Serum levels of the inflammatory alarm protein, high-motility group box 1 (HMGB1), have been reported to be overexpressed in the spinal cord and brain in an ALS mice model and in biopsies from patients with ALS [26, 27]. Because these prevalent damage factors and danger signals are involved in ALS, we hypothesized that two signals belonging to the danger signal family, eosinophil-derived neurotoxin (EDN) and eosinophil cationic protein (ECP), may be correlated with ALS.

EDN, also known as RNase2, is a member of the ribonuclease A superfamily [28]. EDN is a single-chain polypeptide with an observed molecular mass of 18.6 kDa. EDN is expressed mainly in eosinophils but is also detected in mononuclear cells and possibly neutrophils [29]. EDN possesses full ribonucleolytic activity and is involved in defending the upper bronchial tract from infection by respiratory syncytial virus [30, 31]. In addition, EDN is likely to be a host molecule that may induce proinflammatory cytokine production in monocyte/macrophages and the maturation of dendritic cells through Toll-like receptor 2 (TLR2) [32]. Furthermore, EDN causes serious damage to myelinated neurons in the rabbit brain, an event known as the Gordon phenomenon [33–35]. Damage to Purkinje cells and devastating spongiform degeneration in the white matter of the brainstem, cerebellum, and spinal cord are also caused by EDN [36]. Therefore, it is rational to suggest that EDN plays a critical role in neuronal damage and is involved in the loss of neurons, resulting in neurodegenerative disorders. 

ECP is a paralog of EDN in humans, and they share 70% similarity at the protein level. They are both secreted by activated eosinophils during pathogenic stimulation and inflammatory processes [37]. In patients with asthma, the serum level of ECP is elevated and serves as a clinical biomarker for monitoring asthma severity [38]. In pathophysiological conditions, accumulation of ECP induces chronic inflammation and enhances the severity of inflammation such as that which occurs during the inflammation of the intestinal mucosa in Crohn's disease [39]. The tissue damage attributed to ECP depends on its interaction with the organism surface, which occurs during its translocation into the cell. The hypothesized mechanism of ECP-triggered cell damage is that ECP destabilizes the cell membrane via the processes of pore formation, permeability changes, and membrane leakage [40]. 

 Because EDN and ECP participate in the induction of inflammatory diseases and because both serve as disease biomarkers, we examined the serum levels of EDN and ECP in patients with ALS.

## 2. Materials and Methods

### 2.1. Participants

 The demographic information for the normal control individuals and the patients with ALS is given in Table 1. Forty-four patients with ALS (25 male, 19 female), 39 patients with Alzheimer's disease (AD; 16 male, 23 female), 40 patients with Parkinson's disease (PD; 20 male, 20 female), and 44 age-matched, unrelated healthy controls (24 male, 20 female) were recruited by the Taipei City Hospital Zhongxiao Branch, Taipei, Taiwan. Informed consent was obtained before blood sampling, and all procedures were approved by the Institutional Review Board of Taipei City Hospital. All patients and controls were Taiwanese. The clinical severity of patients with ALS was evaluated using the Amyotrophic Lateral Sclerosis Functional Rating Scale-Revised (ALSFRS-R) ranged from 0 to 47. The mean and SD of ALSFRS-R in this study were 17.8 and 13.28, respectively. The disease duration of patients with ALS ranged from 2 to 84 months (mean = 13.6 months, SD = 14.65), and the onset types were classified as brainstem (B), hand (H), and foot (F). 

### 2.2. Enzyme-Linked Immunosorbent Assay (ELISA)

 Venous blood (10 mL) was collected from patients with ALS, AD, and PD as well as controls; all participants were free of acute infection and acute stress at the time of collection. Serum collected using yellow-stopper clot-accelerating tubes was harvested by centrifugation at 3000 ×g for 20 min, divided into aliquots, and frozen at −30°C until use. The serum levels of EDN and ECP were measured using commercially available ELISA kits for human EDN and ECP (both kits from MBL, Naka-Ku, Nagoya, Japan).

### 2.3. Statistical Analysis

 The serum concentrations of EDN and ECP were compared among patients with ALS, AD, and PD and controls using an ANOVA. The one-way ANOVA test was used to determine the difference among all of the test groups. When the *P* value of the ANOVA reaches the significance level, Bonferroni multiple comparison test was used to determine the significant difference among the groups of control, AD, PD, and ALS. The Spearman rank correlation was used to correlate the levels of EDN or ECP versus the subgroups of age, ALSFRS-R, disease duration, or onset types.

## 3. Results

### 3.1. EDN Is Elevated in Sera of Patients with ALS

**Figure 1:**
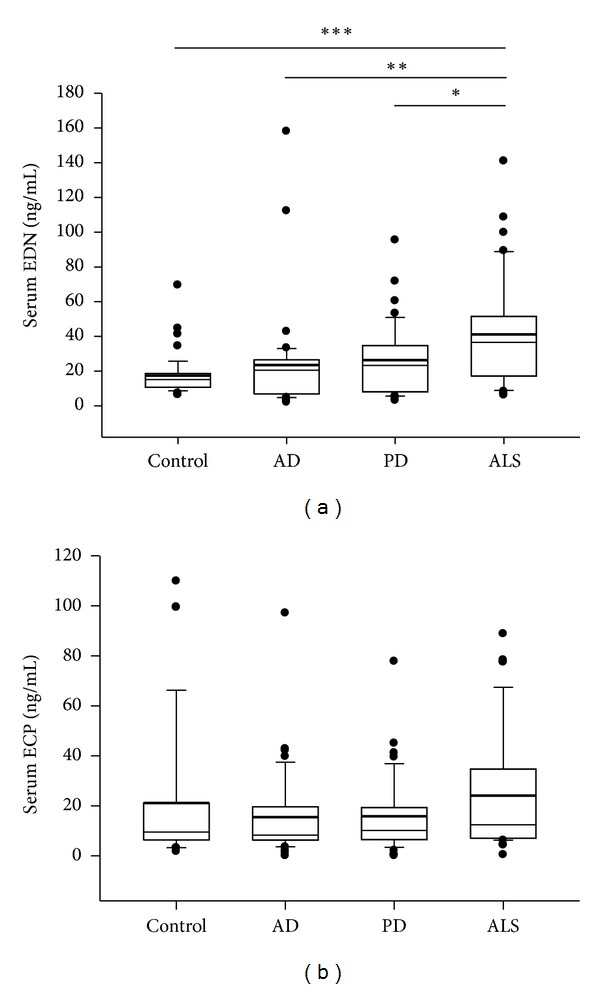
Serum EDN is elevated in patients with ALS. Serum concentrations of EDN (a) and ECP (b) in controls (*N* = 44) and patients with AD (*N* = 39), PD (*N* = 40), and ALS (*N* = 44). *P < 0.05, **P < 0.01, ***P < 0.005. The bold black line indicates the mean.

The concentration of serum EDN in patients with ALS, AD, and PD and in healthy controls was measured using a commercial ELISA kit. The average EDN levels in patients with ALS and controls were 45.7 ng/mL (SD = 29.3 ng/mL; range, 8.9–140.9 ng/mL) and 21.0 ng/mL (SD = 14.9 ng/mL; range, 8.6–79.4 ng/mL), respectively (Table 2). The level of EDN was significantly increased by 2.17-fold in the sera of patients with ALS as compared with the control group (*P* < 0.005; Figure 1(a)). The serum EDN level in patients with ALS was significantly increased 1.61- and 1.84-fold as compared with patients with AD (28.3 ng/mL; SD = 36.6 ng/mL; range, 1.9–158.0 ng/mL) and PD (24.8 ng/mL; SD = 20.7 ng/mL; range, 3.1–95.5 ng/mL), respectively (Figure 1(a)). These data indicate that serum EDN is specifically elevated in patients with ALS and may serve as an indicator for ALS.

Next, the ALSFRS-R, disease duration, age, and disease onset among patients with ALS were analyzed and statistically correlated with EDN levels. The clinical indicators were not correlated with EDN levels (Table 2).

### 3.2. The Serum Levels of ECP Are Similar among Patients with ALS, AD, and PD and Healthy Controls

The levels of ECP were not significantly different in the sera from patients with ALS (24.1 ng/mL; SD = 24.5 ng/mL; range, 0.4–88.8 ng/mL), AD (15.4 ng/mL; SD = 17.4 ng/mL; range, 0–97.1 ng/mL), and PD (15.8 ng/mL; SD = 15.2 ng/mL; range, 0–77.8 ng/mL) and healthy controls (21.1 ng/mL; SD = 27.4 ng/mL; range, 1.7–109.9 ng/mL; Figure 1(b), Table 2). Correlations between ECP levels and clinical indicators were also compared. No meaningful correlations were observed between ECP levels and each indicator. Hence, EDN, but not ECP, may serve as an indicator for ALS.

### 3.3. Prediction of Performance of EDN as an Indicator for ALS

**Figure 2:**
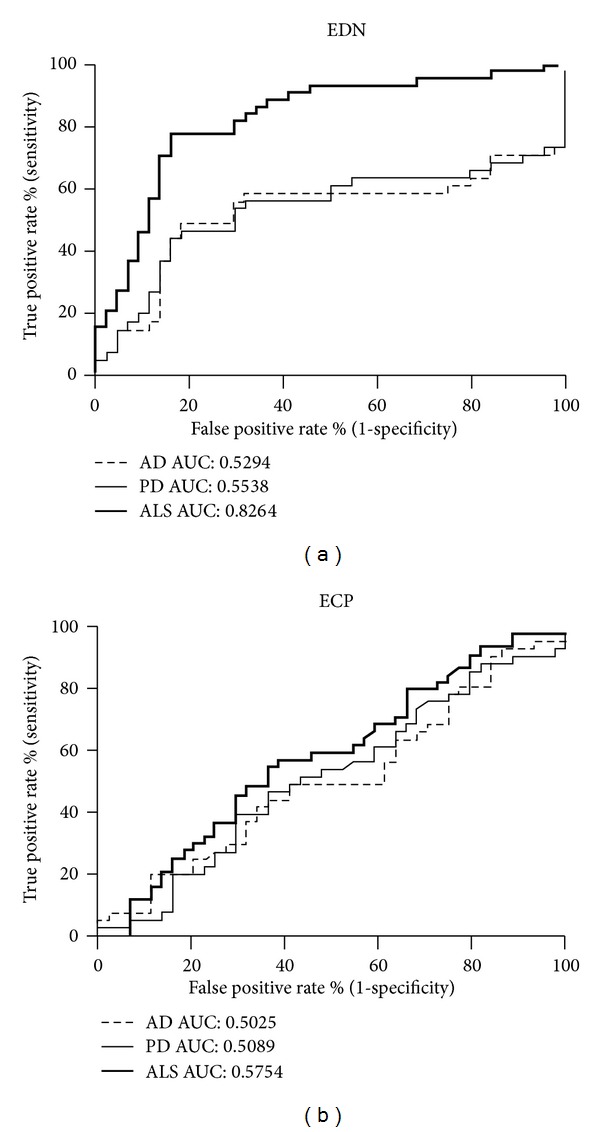
The performance of EDN as an indicator for ALS. ROC curves represent the performance of a prediction method. The curves for EDN (a) and ECP (b) for predicting the neurodegenerative diseases AD, PD, and ALS. The area under the curve (AUC) was calculated.

A receiver operating characteristic (ROC) curve was used to determine the performance of EDN and ECP correlated among ALS, AD, and PD. EDN showed the best performance with 88.53% accuracy, 77.27% sensitivity, and 84.09% specificity when the cut-off concentration was set at 23.43 ng/mL for ALS. ROC curve analysis also showed that EDN had the highest area under the curve (AUC) value of 0.8264. As expected, values for AD and PD were 0.5294 and 0.5538, respectively, indicating a nearly random distribution (Figure 2(a)). For ECP, 54.55% sensitivity, 63.64% specificity, and 69.61% accuracy were detected for predicting ALS. The AUC value for ALS was 0.5754, similar to a random distribution, and AUC values for AD and PD were 0.5025 and 0.5089, respectively (Figure 2(b)). These results indicate that EDN, but not ECP, may serve as an ALS indicator. 

## 4. Discussion

Damage-associated molecular patterns (DAMPs) play an important role in stimulating macrophages and T lymphocytes [41]. Recent studies have indicated that some DAMPs including ROS [42], HSPs [43], and HMGB1 [43] are present or are overexpressed in the spinal cord [44] and motor cortex in SOD1-G93A transgenic mice and/or patients with ALS [45, 46]. In this study, we report that another DAMP, EDN, was elevated in the sera of patients with ALS. EDN is secreted from human activated eosinophils and neutrophils [47] and is a powerful and important neurotoxin that causes neuronal and axonal damage by inducing loss of normal cell shape [33, 48]. Furthermore, the severe spongy vacuolation of the white matter that is seen in the brainstem, cerebellum, and spinal cord in mice is also caused by EDN overexpression [36, 49]. This phenomenon suggests that the eosinophil-secreted protein EDN plays a crucial role in progressive neurodegenerative disorders. Our analyses showed no significant correlation between the serum concentration of EDN and the stage of ALSFRS-R. ALSFRS-R integrates various aspects of the ALS clinical condition including muscle power, control ability, vigor, and voluntary movement [50, 51]. Our finding may suggest that the neuroinflammation resulting from elevated EDN occurs in the early stage of ALS and does not correlate with disease progression. 

We also observed no interaction between the level of EDN and the age of patients with ALS. In the elder population, chronic inflammation increases, and inflammatory stimulation is upregulated during aging [52, 53]. Therefore, we propose that EDN levels correlate with neuroinflammation and may be highly specific to ALS without aging effects. Therefore, EDN may be potentially useful for early diagnosis of ALS caused by peripheral neuroinflammation at any age. 

Neuroinflammation is critical in the pathogenesis of ALS [54]. High numbers of dendritic cells and high levels of monocyte chemotactic protein-1 (MCP-1) are found in the ALS mouse model, which shows neuroinflammation [55, 56]. MCP-1 has been implicated as a chemokine that attracts monocytes, T cells, and dendritic cells [57]. MCP-1 may chemoattract various immunocytes to inflammatory sites and induce more severe neuroinflammation [56, 58]. In ALS, excessive EDN in hyperimmune conditions may be an autoaggressive factor that interacts with normal neurons to damage those cells and destroy their function, similar to autoimmune diseases [59]. Therefore, higher levels of EDN in ALS may be a damage factor, signal, or immune response. Nevertheless, the mechanisms are still unclear. 

Although macrophages, mast cells, and T cells are reported to induce neuroinflammation in the cortex and spinal cord in ALS [60] and although many inflammatory molecules including interleukin-6, interferon-*γ*, tumor necrosis factor-*α*, and nitric oxide are elevated in the serum of patients with ALS [61, 62], no studies have elucidated the roles of EDN in ALS. This study is the first to report a correlation between EDN and ALS, and we propose that EDN may participate in the pathogenesis of ALS and may serve as an ALS indicator. 

## Figures and Tables

**Table 1:** Study population demographics and clinical characteristics.

	*N*	Age (years)^a^			Gender	ALSFRS-R^b^	Disease duration^c^	Type of onset
		Mean (SD)	Range	Median	(M/F)	Mean (SD)	Mean (SD)	(B/H/F)^d^
Control	44	60 (10.10)	30–87	65	23/17	—	—	—
AD	39	80 (7.71)	54–90	81	16/23	—	—	—
PD	40	76 (9.17)	53–90	77	20/20	—	—	—
ALS	44	58 (9.88)	34–79	57	25/19	17.8 (13.29)	13.6 (14.65)	13/12/19

^a^Age was at the time of blood collection.

^b^The ALSFRS-R is a scale from 0 to 48 which assesses disability in patients with motor neuron diseases. 0 means serious and 48 means normal.

^c^The disease duration indicates months since the onset of symptoms.

^d^B: brainstem, H: hand, F: foot.

**Table 2:** The concentrations of EDN and ECP in sera from patients with ALS, AD, and PD and from controls.

Group (*N*)	EDN^a^							ECP^a^						
	mean (SD, range)							mean (range)						
		*P* value							*P* value					
		Control	AD	PD	ALS				Control	AD	PD	ALS		

Control (44)	21.0 (14.9, 8.6–79.4)	—						21.1 (27.4, 1.7–109.9)	—					
AD (39)	28.3 (36.6, 1.9–158.0)	0.23	—					15.4 (17.4, 0–97.1)	0.26	—				
PD (40)	24.8 (20.7, 3.1–95.5)	0.33	0.61	—				15.8 (15.2, 0–77.8)	0.27	0.73	—			
ALS (44)	45.7 (29.3, 8.9–140.9)	**<0.005**	**0.01**	**0.02**	—			24.1 (24.5, 0.4–88.8)	0.06	0.07	0.06	—		

^ b^ALSFRS-R		*P* value							*P* value					
		Control	0–9	10–19	20–29	30–39	40–47		Control	0–9	10–19	20–29	30–39	40–47

0–9 (15)	42.6 (27.6, 11.9–99.9)	0.63	—					18.7 (19.1, 4.3–77.7)	0.60	—				
10–19 (11)	45.9 (37.5, 8.9–140.9)	0.17	0.24	—				25.6 (27.4, 4.3–78.4)	0.43	0.71	—			
20–29 (7)	39.9 (21.1, 14.6–69.7)	0.08	0.59	0.90	—			21.6 (22.5, 6.3–66.3)	0.20	0.50	0.91	—		
30–39 (7)	51.3 (25.8, 17.9–89.3)	0.27	0.35	0.66	0.49	—		26.2 (27.6, 0.4–77.5)	0.35	0.66	0.17	0.96	—	
40–47 (4)	57.4 (37.6, 18.3–108.7)	0.75	0.75	0.33	0.33	0.92	—	40.9 (35.4, 12.1–88.8)	0.42	0.75	0.33	0.33	0.42	—

^ c^Duration		*P* value							*P* value					
		Control	1–12	13–24	25–36	>37			Control	1–12	13–24	25–36	>37	

1–12 (31)	50.3 (32.7, 8.9–140.9)	0.83	—					18.9 (20.3, 4.3–77.4)	0.75	—				
13–24 (8)	31.3 (12.2, 16.6–48.6)	0.13	0.27	—				32.5 (31.2, 4.3–88.8)	0.88	0.70	—			
25–36 (3)	49.0 (15.1, 33.2–50.2)	NA	^ f^NA	NA	—			58.5 (24.1, 31.5–77.7)	NA	NA	0.33	—		
>37 (2)	27.9 (13.2, 18.6–37.3)	NA	NA	NA	NA	—		18.4 (17.1, 6.3–30.5)	NA	NA	NA	NA	—	

^ d^Age		*P* value							*P* value					
		Control	41–50	51–60	61–70	71–80			Control	41–50	51–60	61–70	71–80	

41–50 (7)	58.9 (42.2, 20.9–140.9)	0.30	—					40.7 (30.6, 10.9–78.4)	0.56	—				
51–60 (19)	35.8 (21.2, 8.9–101.8)	0.06	0.49	—				19.3 (21.1, 4.3–77.7)	0.82	0.56	—			
61–70 (13)	53.5 (30.2, 14.6–108.7)	0.48	0.44	0.91	—			65.4 (26.3, 6.4–88.8)	0.72	0.57	0.93	—		
71–80 (5)	45 (28.1, 16.6–83.4)	0.45	0.95	0.45	0.35	—		9.9 (7.8, 0.4–20)	0.78	0.45	0.68	0.35	—	

^ e^Onset type		*P* value							*P* value					
		Control	Brainstem	Hand	Foot				Control	Brainstem	Hand	Foot		

Brainstem (13)	42.4 (25.2, 14.6–99.9)	0.89	—					19.8 (21.9, 4.3–77.7)	0.54	—				
Hand (12)	51.1 (30.9, 8.9–108.7)	0.68	0.54	—				22.9 (28.7, 6.2–77.5)	0.90	0.86	—			
Foot (19)	44.7 (32.3, 11.9–140.9)	0.92	0.88	0.62	—			27.7 (20.4, 0.42–88.8)	0.39	0.46	0.39	—		

^a^Concentrations are expressed as ng/mL.

^b^ALSFRS-R is a score from 0 to 48 which assesses disability in patients with motor neuron diseases. 0 means serious, and 48 means normal.

^c^Categories are based on the duration of symptoms (months).

^d^Categories indicate age at time of blood collection (years).

^e^Categories indicate the type of onset.

^f^The NA were divided according to the unanalyzable group (sample number of subgroup <3).

The one-way ANOVA test and Bonferroni multiple comparison test were used to determine the significance among the test groups of control, AD, PD, and ALS.

The Spearman rank correlation test was used to correlate the levels of EDN and ECP versus the subgroups of age, ALSFRS-R, duration, and onset types.
